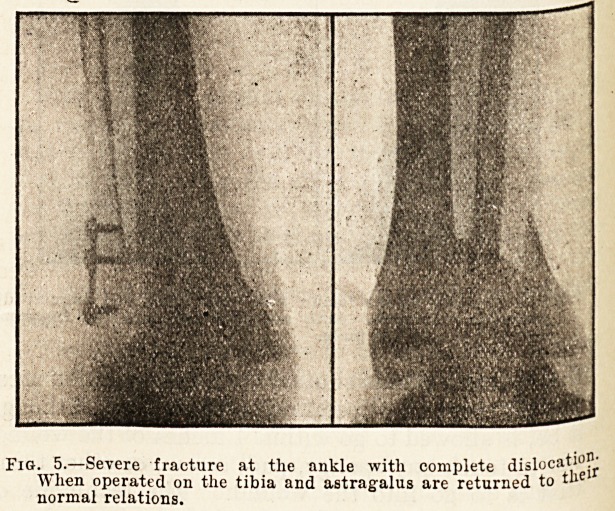# The Operative Treatment of Fractures

**Published:** 1911-01-14

**Authors:** 


### Surgery.

### THE OPERATIVE TREATMENT OF FRACTURES.

### II.—Simple Fractures.

The treatment by operation in this class of injury may be summed up thus: "Asepsis, again asepsis—and always asepsis." In operations on the abdomen there is a margin left which saves the surgeon from disaster when his asepsis is at fault. In operations on bones there is no such margin; there is no tissue except, perhaps, synovial membrane, in which the immunity special to the tissue is so low.

### Mr. Lane's Method.

The principles of technique used by Mr. Lane will be described here. After a thorough preliminary cleansing with soap and water and compressing over night, the skin area is well washed with a solution of biniodide of mercury in spirit, and then everything but the area of incision is shut off with towels. The incision is next made, and nothing further is done until pieces of sterile lint have been pinned to the edge of the wound. In this way the whole of

Fig. 1.—Fracture of the femur, with shortening and angular displacement outwards. Both are corrected, and the fragments kept in place by the plate.

the patient's skin is shut off from the operation area. For the rest of the operation no finger, gloved though it be, is allowed to go within 4 inches of the wound, nor to touch any sponge, pad, screw, or other thing that is to go into the wound. The technique of asepsis has been described first because it dominates all other points. Before the operation $x$-ray photos are taken in two planes; any two planes will do if the angle between them is known, but at right angles to one another is most convenient for practical purposes. If this be done, there is no need for stereoscopic photos, and the former have the advantage that they can easily be brought into the theatre and referred to without the need of any special apparatus. To do these operations properly, a special set of bone levers and forceps are needed. Some surgeons deny this and say the operations can well be done without. Anyone who has seen a surgeon operate with the forceps used by Mr. Lane, and has compared this with another surgeon doing a similar operation without them, will readily

Fig. 2.—Fracture of the neck of the humerus, with perfect apposition. By ordinary retentive apparatus it is practically impossible to keep control of the short upper piece.

recognise the advantage of these instruments. The plates used by Mr. Lane are made of steel with three or more holes for screws. The screws, whose thread

Fig. 3.—A fracture of the humerus, by reposition the sharp projections of bone cannot damage the musculo-spiral nerve. Owing to the exact apposition no provisional callus is formed, and again the nerve is protected.

passes right up to the head, go through one layer of compact bone only.

### Indications.

Having briefly sketched the technique of these operations, the indications remain to be described.

The first indications to be considered are the surgeon and his surroundings; if the surgeon has not the opportunity to practise a technique as rigid as that described above, he should not attempt to operate on a simple fracture. An argument that used to be brought against this treatment was that it could never be justifiable to convert a simple fracture into a compound one with the attendant risks of sepsis. This argument is unassailable, and if any surgeon in cutting down on a fracture admits those risks of sepsis that occur in a compound fracture, then he should not cut down. No one has ever advocated this treatment as being applicable to attendance in a country cottage or a London tenement.

### Weighing the Risks.

But even when all the conveniences of an up-to-date hospital are to hand, there is still another

Fig. 4.—Comminuted fracture of the tibia with fracture of the fibula. By operation the comminuted piece is returned to its exact position.

question to be decided. Is the advantage to be gained from the improved result greater than the increased risk run by the patient?

There are three factors to be considered here: the local condition of the fracture; the amount of use that the patient wants to have in the limb; and the general condition of the patient.

### Practical Considerations.

With regard to the last two, the large majority of fracture cases occur in the active period of life. In practically every sphere of activity a man's wage-earning capacity is impaired by a partial disuse of any limb, and in the labouring classes it is entirely dependent upon the integrity of all four limbs. In all these cases the increased danger of an operation by a skilled surgeon is as nothing compared with the advantage to be gained from a perfect limb. In a high state of civilisation this point is rather apt to be minimised. There are so many instances where the brain has triumphed over the body, Macaulay's picture of "the asthmatic skeleton" facing "the hunch-backed dwarf" is so vivid and striking that one is prone to forget that these cases are the exception, and that the multitude of both men and women are as dependent nowadays upon the integrity of their limbs as they were in a less highly civilised state.

### The Importance of Discrimination.

On the other hand, if the condition of the patient is such that the risk of the operation is increased, or the advantage to be gained is lessened, there is an indication not to operate. On the one hand, are those patients in whom the repair of the tissues is lowered, such as cases of renal disease, and all conditions where an anæsthetic is dangerous, such as diabetes or bad bronchitis; on the other hand are the aged who have to lead but a vegetative existence for the rest of their lives.

Again, there are certain patients whose bones are so brittle that they scrunch when a pair of forceps is applied and that no screw will hold in them. These are usually aged people or those addicted to alcoholism. Turning to the other factor, the local condition of the fracture, there is and there probably always will be, a great divergence of opinion as to which fractures need operation, and which will unite in such perfect apposition that no operation is needed. There is probably no one who will deny that the best way to treat a fracture of the surgical neck of the humerus with dislocation of the upper piece is by operation. There is, on the other hand, no surgeon who would cut down on a fracture of the tibia that showed no displacement. But where the dividing line comes is capable of much discussion. It may be said,

Fig. 5.—Severe fracture at the ankle with complete dislocation. When operated on the tibia and astragalus are returned to their normal relations.

however, that those who thoroughly realise the causes of loss of function indicated in the first paper, and who consider that perfect apposition of the broken parts is the ideal to be aimed at, will be more inclined to operate than those who believe that a useful limb is frequently obtained with a bad "cosmetic" result.

(*To be continued.*)

[We are indebted to Mr. W. Arbuthnot Lane for permission to reproduce these photographs, which were taken from his cases.—Ed. The Hospital.]

## Figures and Tables

**Fig. 1. f1:**
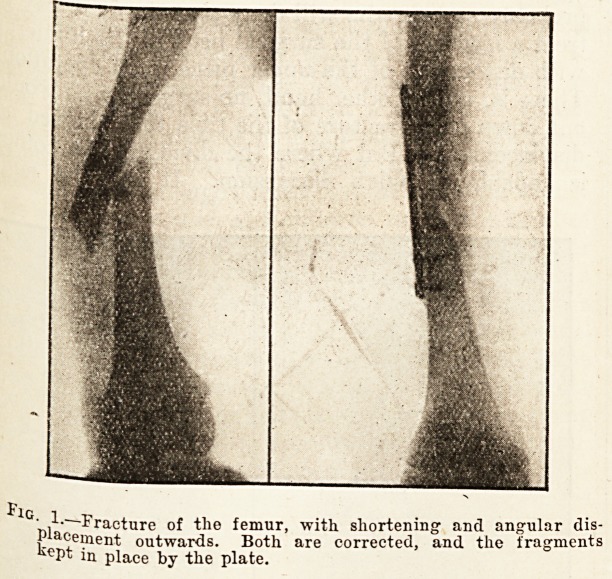


**Fig. 2. f2:**
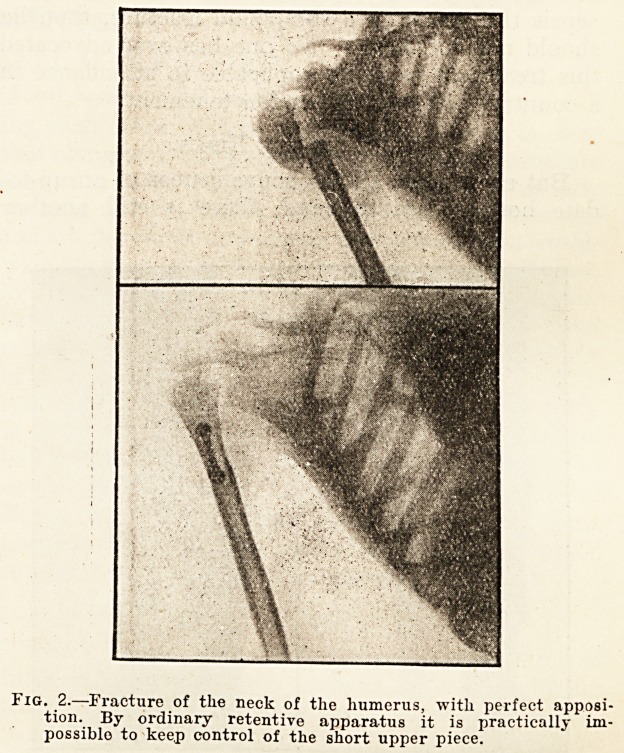


**Fig. 3. f3:**
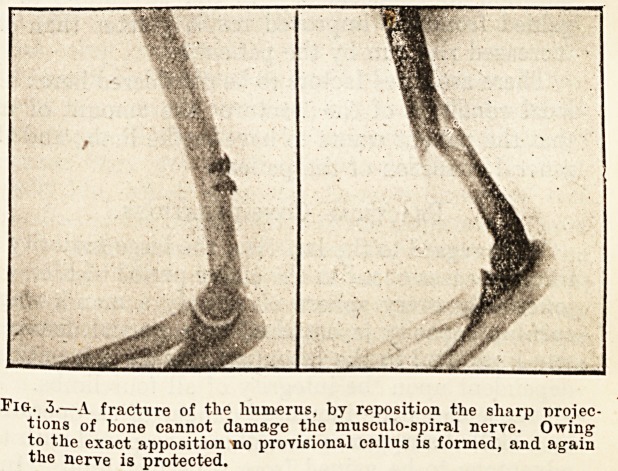


**Fig. 4. f4:**
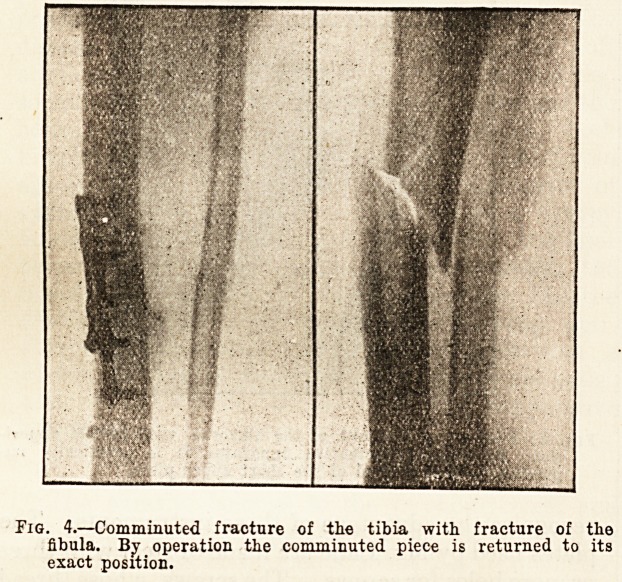


**Fig. 5. f5:**